# Longitudinal association between self‐compassion and intuitive eating: Testing emotion regulation and body image flexibility as mediating variables

**DOI:** 10.1002/jclp.23569

**Published:** 2023-07-18

**Authors:** Mariel Messer, Sohee Lee, Jake Linardon

**Affiliations:** ^1^ School of Psychology Deakin University Geelong Victoria Australia; ^2^ Faculty of Health and Environmental Science Auckland University of Technology Auckland New Zealand; ^3^ Center for Social and Early Emotional Development and School of Psychology Deakin University Burwood Victoria Australia

**Keywords:** body image, emotion regulation, intuitive eating, self‐compassion

## Abstract

**Objectives:**

Growing evidence suggests that intuitive eating is associated with numerous positive mental health and well‐being constructs. Understanding factors that predict intuitive eating is necessary for identifying practical targets to enhance this style of eating, yet research identifying such predictors is scarce. Self‐compassion is one variable that could enhance intuitive eating because it involves the practice of healthy emotion regulation skills that may disrupt the tendency to turn to food to cope during distressing situations. The present study tested for a longitudinal association between self‐compassion and intuitive eating. We also tested whether this association was mediated by indices of emotion regulation (i.e., global emotion regulation skill scores and body image flexibility).

**Method:**

Adult women (*n* = 3039) were invited to completed study measures at baseline (T1), 4‐month follow‐up (T2), and 8‐month follow‐up (T3). Path analyses were computed to test hypothesized indirect effects.

**Results:**

A direct path from T1 self‐compassion to T3 intuitive eating emerged, such that higher self‐compassion levels predicted increased intuitive eating over time. However, this association was not mediated by T2 emotion regulation skills nor body image flexibility.

**Conclusion:**

Findings suggest that self‐compassion may facilitate an intuitive eating style, which does not appear to be explained by certain emotion regulation skills.

## 1 INTRODUCTION

Over the past decade, a considerable amount of research has focused on understanding adaptive eating patterns. One proposed adaptive eating pattern that has received substantial research attention during this time is intuitive eating, which is defined as having a strong connection to biological cues and eating in response to these cues (Tylka, 2006). People who eat intuitively do not obsess over food or dieting, assign moral categories to foods (“good” vs. “bad”), eat to soothe emotional experiences, nor ignore their biological hunger cues. Rather, they opt for foods they enjoy while still ensuring their body functions optimally, rely on internal rather than external cues to determine when and how much to eat, and respect their satiety signals by refraining from eating when they are comfortably full (Tylka & Kroon Van Diest, 2013).

Accumulating evidence is beginning to highlight the potential mental health benefits of intuitive eating. Prospective studies show higher baseline intuitive eating scores to be associated with reduced odds of engaging in eating disorder behaviours, fewer body image problems, higher self‐esteem, and decreased depressive symptoms (Christoph et al., 2021; Hazzard et al., 2020; Linardon, 2021, 2022; Messer et al., 2021). Furthermore, pilot studies of interventions designed to teach intuitive eating principles have reported improvements in several key indicators of mental health and well‐being (Babbott et al., 2023; Burnette & Mazzeo, 2020; Bush et al., 2014). Combined, available evidence indicates that promoting an intuitive eating style may be an important target for intervention.

Understanding factors that predict intuitive eating is necessary for identifying potential practical targets to enhance this style of eating. As it stands, few longitudinal studies have been designed to understand predictors of intuitive eating; however, preliminary evidence suggests that both positive and negative body image components are potentially important predictors of higher and lower intuitive eating levels over time, respectively (Linardon, 2022; Messer et al., 2021). Additional longitudinal research that identifies other factors predictive of intuitive eating is needed to broaden our understanding of this construct.

One variable that may enhance intuitive eating is self‐compassion. Self‐compassion involves displaying a caring relationship with the self, particularly in instances of perceived failure, inadequacy, and personal suffering (Gilbert, 2014). According to Neff (2003), self‐compassion is comprised of three main components that each contain a positive and negative pole that represents compassionate versus uncompassionate ways of responding. These include self‐kindness versus self‐judgement, a sense of common humanity versus isolation, and mindfulness versus over‐identification.

Given this operationalization, some contend that self‐compassion is optimally suited to cultivate an intuitive eating style for two main reasons (Schoenefeld & Webb, 2013). First, self‐compassion invokes a trait‐like attitude of understanding and patience directed at the self, which may translate to being more caring toward oneself when experiencing negative thoughts, feelings, or beliefs. Practicing such self‐kindness may prevent the person from acting on these adverse experiences through impulsive eating behaviours (e.g., binge eating), and may also encourage the person to treat their body with respect by honouring its physiological cues. Second, self‐compassionate individuals are capable of attending to present‐moment experiences with an attitude of non‐judgemental acceptance. Thus, when faced with self‐critical thoughts, this attitude might help the person let go of any habitual or harmful responses and instead select more adaptive ways of responding, such as by nourishing the body with nutritious foods or opting for foods that align with their bodies' needs. Even though there is good reason to believe that self‐compassion may cultivate intuitive eating, it remains unclear whether self‐compassion predicts intuitive eating patterns over time.

There is a growing body of research linking self‐compassion with intuitive eating. Recent meta‐analytic research has reported a correlation of 0.41 between the two constructs (Linardon, Tylka, et al., 2021), suggesting that those who score high on self‐compassion also score high on intuitive eating. However, given that available research correlating these constructs is based on cross‐sectional designs, the temporal order of this relationship remains poorly understood. Furthermore, if a temporal relationship exists, the mediational mechanisms accounting for this association have also yet to be identified.

Emotion regulation skills may, in part, explain how, why, or through what mechanisms self‐compassion leads to intuitive eating. That is, practicing self‐compassion may improve the ability to identify and accept emotions, alleviate emotional avoidance, diminish hyperarousal, and reappraise self‐critical beliefs (Diedrich et al., 2014, 2016). Skills like these, in turn, may disrupt the tendency to use food as a coping mechanism (Haedt‐Matt & Keel, 2011), enabling trust in hunger and satiety cues. Previous research has established robust positive relationships between self‐compassion, intuitive eating, and adaptive emotion regulation skills (Inwood & Ferrari, 2018; Linardon, Tylka, et al., 2021). Similarly, specific emotion regulation skills in the context of body image may also explain how and why self‐compassion could cultivate intuitive eating. Being kind to oneself may help individuals embrace negative automatic thoughts, feelings, or sensations about the body without acting on or trying to change them (a process coined body image flexibility; Sandoz et al., 2013). People who exhibit traits of body image flexibility behave in ways that are consistent with their values, which might involve nourishing the body with what it needs and desires, despite experiencing continued threats to their appearance (e.g., pressures to diet). In light of established bivariate relationships between self‐compassion, body image flexibility, and intuitive eating (Linardon, Anderson, et al., 2021), it is possible that this domain‐specific emotion regulation skill also mediates the association between self‐compassion and intuitive eating.

The present study had two broad aims: the first is to test whether self‐compassion is directly and temporally associated with intuitive eating levels over time. The second is to test whether the relationship between self‐compassion and intuitive eating (if present) is mediated by general emotion regulation skills and the domain‐specific emotion regulation skill of body image flexibility. We hypothesized that the direct temporal relationship between self‐compassion and intuitive eating will be mediated by emotion regulation and body image flexibility scores.

## 2 METHOD

### 2.1 Participants and procedure

Participants were invited to complete an online questionnaire battery as part of a larger study at baseline (T1), 4‐month follow‐up (T2), 8‐month follow‐up (T3). Study flyers were advertised on the homepage of the authors' website and Instagram account that provides general information about eating behaviours and body image patterns. A full description of this website, including its content and audience, can be found elsewhere (Linardon, Rosato, et al., 2020). Study advertisements indicated that the researchers were interested in understanding factors associated with adaptive and maladaptive eating patterns. Anyone could participate so long as they were aged 18 years or over and could understand English language. Participants were not compensated for completion of surveys.

Of the 3163 participants recruited at T1, only 124 were men (with <25% of men with complete data at T3). Given that the number of men was too low to conduct gender‐stratified analyses, and since gender differences in self‐compassion (Yarnell et al., 2015) and intuitive eating (Linardon, Tylka, et al., 2021) exist, a decision was made to exclude men from the subsequent analyses. The final sample was 3039 adult women (*M*_age_ = 32.3 years, *SD* = 8.48), most of which were White (76.4%). Other ethnicities reported were Asian (10.2%), Hispanic (5.6%), mixed race (4.1%), African American (0.7%), Pacific Islander (0.3%), Native American (0.1%), and “other” (2.7%). All participants provided informed consent and ethics approval was obtained from Deakin University.

### 2.2 Measures

Table 1 presents the reliability estimates of each measure.

**Table 1:** Correlations between study variables.

Variable	1	2	3	4	5	6	7	8
1. T1 self‐compassion								
2. T1 body image flexibility	0.55							
3. T1 emotion dysregulation	−0.67	−0.54						
4. T1 intuitive eating	0.51	0.62	−0.42					
5. T2 body image flexibility	0.51	0.80	−0.49	0.60				
6. T2 emotion dysregulation	−0.57	−0.42	0.76	−0.38	−0.49			
7. T2 intuitive eating	0.44	0.52	−0.38	0.82	0.63	−0.42		
8. T3 intuitive eating	0.47	0.50	−0.39	0.78	0.58	−0.40	0.85	
Mean	2.70	20.02	41.01	3.01	21.51	39.06	3.11	3.14
Standard deviation	0.76	8.23	15.27	0.74	8.12	14.56	0.77	0.74
*α*	0.87	0.92	0.95	0.91	0.93	0.94	0.92	0.91
All correlations significant at *p* < 0.001								

#### 2.2.1 Self‐compassion

Self‐compassion was assessed using the 12‐item Self‐Compassion Scale Short‐Form (SCS‐SF; Raes et al., 2011). The 12 items are rated along a 5‐point scale, ranging from 1 (*strongly disagree*) to 5 (*strongly agree*). After reverse scoring the relevant items, the average score is taken to produce a total score, with higher scores reflecting higher self‐compassion levels. The SCS‐SF has a near perfect correlation with the full‐form and has demonstrated internal reliability and construct validity (Raes et al., 2011).

#### 2.2.2 Body image flexibility

Body image flexibility was assessed via the abbreviated 5‐item version of the Body Image Acceptance and Action Questionnaire (BI‐AAQ; Sandoz et al., 2013). The five items are rated on a 7‐point scale ranging from 1 (*never true*) to 7 (*always true*), are reverse scored, and then summed to produce a total score. Higher scores reflect higher levels of body image flexibility. The abbreviated BI‐AAQ has exhibited a unidimensional structure, adequate internal consistency, evidence of convergent validity, and was shown to explain 96% of the variance in the data of the original version, having an almost perfect correlation with it (Basarkod et al., 2018; Linardon, Messer, et al., 2020).

#### 2.2.3 Emotion regulation

Emotion regulation was assessed using the abbreviated 16‐item Difficulties in Emotion Regulation Scale (DERS‐16; Bjureberg et al., 2016). The DERS‐16 is a self‐report measure that assesses multiple dimensions of emotion dysregulation. Participants were asked to rate the extent to which each item applies to them on a 5‐point scale, ranging from 1 (*almost never*) to 5 (*almost always*). Items were summed to produce a total score, with higher scores reflecting greater deficits in emotion regulation. Evidence of internal consistency, test−retest reliability, and convergent validity of the DERS‐16 has been established (Bjureberg et al., 2016).

#### 2.2.4 Intuitive eating

Intuitive eating was assessed via the 23‐item Intuitive Eating Scale‐2 (Tylka & Kroon Van Diest, 2013). Items are rated along a 5‐point scale ranging from 1 (*strongly disagree*) to 5 (*strongly agree*) and are averaged to produce a total score. Higher scores reflect greater levels of intuitive eating. The internal consistency, 3‐week test–retest reliability, and construct validity have been supported (Tylka & Kroon Van Diest, 2013).

### 2.3 Statistical analyses

Assumption testing, descriptive statistics, and attrition analyses were performed using SPSS Version 22, and main analyses were performed using Mplus Version 8.3 (Muthén & Muthén, 2010). A path analysis using the maximum likelihood estimator was conducted to test whether the hypothesized relationship between T1 self‐compassion and T3 intuitive eating was mediated by deficits in emotion regulation and body image flexibility scores at T2. The path model controlled for deficits in emotion regulation, body image flexibility, and intuitive eating scores at T1, and intuitive eating scores at T2. Model fit was evaluated using the root mean square error of approximation (RMSEA), comparative fit index (CFI), and Tucker−Lewis index (TLI). Acceptable model fit was determined through RMSEA values ≤0.08 and CFI and TLI values ≥0.95 (Marsh et al., 2005). Bootstrapping procedures were applied to test for the hypothesized indirect effects. Mplus created 1000 bootstrap samples from the data set by random sampling with replacement, generating the indirect effect and bias‐corrected confidence internal (CI). Indirect effects are considered significant when the 95% CI does not include zero. Full information maximum likelihood was used to account for missing data (Enders & Bandalos, 2001), allowing all participants to be included in the analyses. Standardized path estimates are reported throughout.

## 3 RESULTS

### 3.1 Preliminary analyses

Key assumptions of the general linear model were met. Residuals conformed to a normal distribution (per visual inspection of the histogram), there was no evidence of nonlinearity between predictors and outcomes, nor evidence of heteroscedasticity, error inflation due to multicollinearity (all VIF and tolerance levels well within the cut‐point of >10 and <0.10, as recommended by Field (2013), or influential cases that changes regression coefficients in path models (all Cooks distances <1.0, per Field, 2013).

The percentage of missing data ranged from 0% to 11% at T1, 52%−54% at T2, and 56% at T3. Only 30% (*n* = 889) participants completed all study measures at all three time‐points. Dropouts were compared to completers at T2 and T3 on baseline variables. T2 dropouts were younger (*p* < 0.001, *d* = 0.21) and reported lower body image flexibility scores (*p* = 0.002, *d* = 0.11) than completers, while T3 dropouts were also younger (*p* < 0.000, *d* = 0.15), reported lower body image flexibility scores (*p* < 0.001, *d* = 0.15), and higher deficits in emotion regulation scores (*p* = 0.001, *d* = 0.13), although effect sizes were small in magnitude. Means, standard deviations, correlations, and Cronbach's *α* estimates for study variables across all time‐points are presented in Table 1.

### 3.2 Main analyses

**Figure 1:**
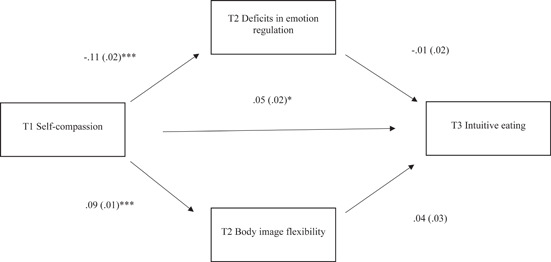
Longitudinal path model testing whether T2 body image flexibility and deficits in emotion regulation mediates the relationship between T1 self‐compassion and T3 intuitive eating. Note that path models controlled for T1 body image flexibility, T1 deficits in emotion regulation, and T1 and T2 intuitive eating. ***p < 0.001, *p < 0.05.

Figure 1 presents the longitudinal mediation model, along with standardized path estimates. The model fit the data well (CFI = 0.99, TLI = 0.97, RMSEA = 0.05). A significant direct path between T1 self‐compassion and T3 intuitive eating emerged, such that higher self‐compassion scores predicted increased intuitive eating scores over time. T1 self‐compassion scores also predicted increased T2 body image flexibility scores and decreased T2 deficits in emotion regulation scores. However, the association between T1 self‐compassion and T3 intuitive eating scores was not mediated by T2 body image flexibility (*b* = 0.00, 95% CI = −0.00 to 0.01) and deficits in emotion regulation scores (*b* = 0.00, 95% CI = −0.01 to 0.01).

## 4 DISCUSSION

The present study tested for a hypothesized longitudinal relationship between self‐compassion and intuitive eating, and whether emotion regulation skills and body image flexibility were mediators of this relationship. Evidence of a direct effect between self‐compassion and intuitive eating was found: higher self‐compassion scores at baseline predicted increased intuitive eating scores at 8 month follow‐up. The effect size for this direct effect was small, suggestive of other factors potentially exerting a stronger influence on intuitive eating. However, contrary to expectations, this association was not mediated by deficits in emotion regulation and body image flexibility scores at 4‐month follow‐up. Overall, present findings could suggest that self‐compassion may facilitate an intuitive eating style, at least to a small extent, but does not appear to do so via certain emotion regulation skills.

The present findings may be interpreted in a number of ways. One explanation could be that emotion regulation skills are only relevant explanatory variables for when self‐compassion is paired with maladaptive (rather than adaptive) eating behaviours. Indeed, there is a large body of evidence showing that better emotion regulation skills and higher body image flexibility scores are prospectively linked with fewer eating disorder behaviours in both treatment‐seeking and community samples (Bodell et al., 2019; Lavender et al., 2015; Linardon, 2021; McClure et al., 2022; Wallace et al., 2014). This is in contrast to evidence linking emotion regulation abilities with intuitive eating, which is limited and only based on cross‐sectional designs (Cardoso et al., 2020). Considering this, it is possible that emotion regulation is not an important underlying mechanism linking self‐compassion to intuitive eating, but instead might only be critical for explaining the well‐replicated link between [low] self‐compassion and eating disorder behaviours (Turk & Waller, 2020).

Another possible explanation for not detecting any mediation effects could be because we operationalized these constructs as trait‐like characteristics and only focused on between‐person relationships. However, these constructs vary not only between persons but also within a person. For instance, experience sampling studies show that around 40% of the variance in students' self‐compassion levels occur within‐persons, suggesting that there are large fluctuations in a person's level of self‐compassion from 1 day to the next (Breines et al., 2014; Kelly & Stephen, 2016). Furthermore, there is also evidence to suggest individuals are more likely to report use of healthy emotion regulation strategies on days where they are more self‐compassionate than usual (e.g., Mey et al., 2023). Such within‐persons associations may subsequently influence aspects of the individual's eating patterns, such as the type and amount of foods consumed, or awareness of physical from emotional hunger. It is necessary for future research to use experience sampling methodology or other intensive time‐series designs to test these mediational pathways at the within‐persons level to gain a better understanding of the role of self‐compassion and emotion regulation skills on adaptive eating behaviours.

This study should be interpreted within the context of its limitations. First, given the correlational nature of this study and the potential for unexplained third variables, causal inferences cannot be made. Randomized experiments that seek to manipulate self‐compassion and emotion regulation skills are required for making causal claims. Second, we sampled mostly White adult women, which limits the generalizability of these findings. It is possible that emotion regulation skills play a more influential role in this relationship among people of different races, genders, or age groups. We recommend replicating these findings in a sample representative of the wider population. Third, there were many dimensions of emotion regulation not assessed in the present study (e.g., negative urgency, problem solving, cognitive reappraisals etc.), but could have emerged as potential mediating variables and thus warrant consideration in future research. Fourth, as with all online research surveys, the possibility of unreliable response patterns due to “bots” remains. Although we took several steps to ensure that the data were reliable (e.g., checked for duplicate IPs and unusual response patterns and completion times), it would have been desirable to have followed prior recommendations put forth by others for ensuring high data quality when conducting online research (Burnette et al., 2022). Fifth, the rate of attrition was high, with only 30% completing all study measures at each time‐point. We found evidence of attrition bias, with drop‐outs reporting poorer emotion regulation (general and body image‐specific). Perhaps no mediational effects were observed because of restricted variability in scores on these measures. That is, retaining participants with poor emotion regulation scores to begin with may have led to more variability and thus a greater ability to detect mediational pathways. Therefore, effective retention strategies in longitudinal designs are needed to prevent the possibility of attrition impacting the results.

## 5 CONCLUSION

In conclusion, the present study identified a small, but direct path between higher self‐compassion scores and increased intuitive eating over 8 months. This association, however, was not mediated by emotion regulation skills or body image flexibility. Consideration of other plausible mediators of this relationship warrants evaluation in future research designs, as this may help to broaden our understanding around how to facilitate an intuitive eating style. Present findings broadly suggest that intuitive eating may be better cultivated through practice of skills known to enhance compassion, kindness, and self‐love.

### 1 PEER REVIEW

The peer review history for this article is available at https://www.webofscience.com/api/gateway/wos/peer-review/10.1002/jclp.23569.

## Data Availability

The data that support the findings of this study are available from the corresponding author upon reasonable request.
